# A Brief Assessment of Patient Safety Culture in Anesthesia and Intensive Care Departments

**DOI:** 10.3390/healthcare11030429

**Published:** 2023-02-02

**Authors:** Andrea Kazamer, Radu Ilinca, Anda Nitu, Ana-Maria Iuonuț, Serban-Ion Bubenek-Turconi, Gerald Sendlhofer, Maria Greabu, Iulia-Ioana Stanescu-Spinu, Daniela Miricescu, Ionela Ganea, Daniela Ionescu

**Affiliations:** 1 CREST Association, 48 Alexandru Odobescu Street, 440069 Satu Mare, Romania; 2 Department of Anaesthesia and Intensive Care I, Iuliu Hatieganu University of Medicine and Pharmacy, 8 Victor Babes Street, 400347 Cluj-Napoca, Romania; 3 Discipline of Medical Informatics and Biostatistics, Faculty of Dentistry, Carol Davila University of Medicine and Pharmacy, 8 Eroii Sanitari Street, 050474 Bucharest, Romania; 4 Department of Psychology, University of Wales, Bangor LL57 2DG, UK; 5 Department of Nursing, Iuliu Hatieganu University of Medicine and Pharmacy, 8 Victor Babes Street, 400347 Cluj-Napoca, Romania; 6 Department of Anaesthesia and Intensive Care, Carol Davila University of Medicine and Pharmacy, 8 Eroii Sanitari Street, 050474 Bucharest, Romania; 7 “Prof. Dr. C.C. Iliescu” Emergency Institute of Cardiovascular Diseases, 1st Department of Cardiovascular Anaesthesia and Intensive Care, 022322 Bucharest, Romania; 8 Research Unit for Safety and Sustainability in Healthcare, c/o Division of Plastic, Aesthetic and Reconstructive Surgery, Department of Surgery, Medical University of Graz, 2, 8036 Graz, Austria; 9 Discipline of Biochemistry, Faculty of Dentistry, Carol Davila University of Medicine and Pharmacy, 8 Eroii Sanitari Street, 050474 Bucharest, Romania; 10 Department of Modern Languages, Faculty of Medicine, Carol Davila University of Medicine and Pharmacy, 8 Eroii Sanitari Street, 050474 Bucharest, Romania; 11 Outcome Research Consortium, Cleveland, OH 44195, USA

**Keywords:** patient safety, HSOPSC, anesthesia and intensive care

## Abstract

Due to the nature of their activity, anesthesia and critical care have generally well-developed patient safety cultures, which are linked to a greater level of incident awareness and reporting during clinical activity. In order to determine the status quo and identify and adopt, where appropriate, techniques and instruments for further improving patient safety, it is necessary to evaluate the culture and barriers in these departments. The main objective of our study was to assess patient safety culture in Romanian anesthesia and intensive care departments (AICDs), to pinpoint the areas that may need improvement, and to examine the correlation between the prevalence of adverse event reporting, as well as the level of self-reported patient safety culture. To determine how anesthesia and intensive care department staff perceived patient safety, the Hospital Survey on Patient Safety Culture (HSOPSC) was used in a translated Romanian version. In total, 1200 employees from 36 anesthesiology and intensive care departments across 32 hospitals in Romania received the questionnaire, representing 42.66% of all anesthesia and intensive care departments in the country. In 7 of the 12 examined dimensions, significant differences between tertiary and secondary hospitals were observed. Among all dimensions, the highest positive score was for “organizational learning and continuous development”. In general, our study revealed a positive view on patient safety in anesthesia and intensive care departments. Further studies are required to determine a threshold of the level of culture development.

## 1. Introduction

Strengthening patient safety culture as a component of organizational culture to “continually seek to minimize patient harm that may result from caregiving processes” [1] is a widespread goal of healthcare organizations. This effort involves an organization’s staff to maintain a constant and active awareness of which “things do not work”, and thereby recognize mistakes and continuously learn from errors [2].

Anesthesia and intensive care represent one of the most challenging medical specialties, as demonstrated during the COVID pandemic. Treating critical patients generates clinical activities with the risk of side-effects, complications, and life-threatening situations. Consequently, the culture of patient safety in anesthesia and intensive care is of tremendous importance for all stakeholders, namely healthcare workers; the management of an organization; and patient interaction, which influences organizational development.

Promoting the concept of safety in anesthesia and intensive care is supported by the European Society of Anaesthesiology and Intensive Care, which, in 2010, released the Helsinki Declaration on Patient Safety in Anaesthesiology (HD), which was signed by several organizations such as the Board of ESAIC (European Society of Anaesthesiology and Intensive Care), the European Board of Anesthesiology—UEMS, and 33 national societies of anesthesia and intensive care [3]. The HD was immediately endorsed by the World Health Organization (WHO), the World Federation of Societies of Anaesthesiologists (WFSA), and the European Patients’ Federation (EPF). Two recent studies demonstrated that numerous aspects concerning patient safety, which were also suggested by HD, have already been implemented in at least 38 European countries; this is still an emerging process [4,5].

Additionally, in 2019, the Romanian Society of Anesthesia and Intensive Care (SRATI) supported the adoption of the national legislation on patient safety in anesthesiology in accordance with HD principles. They highlighted that creating a culture for patient safety, reporting incidents and adverse events, and debriefing are the most important issues in need of further progress. As in other countries, the critical incident reporting system is promoted by the authority in Romania (National Authority for Quality Management of Health Services (ANMCS)).

Developing a patient safety culture is an evolving process which requires continuous measurement of the efficiency of implemented tools and its effect on environmental culture and factors [6]. So far, to the best of our knowledge, there is no study available that assesses the level of patient safety culture in anesthesia and intensive care departments (AICDs) in Romanian hospitals. Therefore, the main objective of our study was to investigate the safety culture in AICDs from the staff’s perspective. This will further raise awareness of the topic among medical staff [7,8]. The evaluation of safety culture in AICDs will support organizational development by identifying areas for improvement. Furthermore, we aimed to identify differences between hospitals.

This study is the baseline for future benchmarks and actions in improving patient safety in AICDs.

## 2. Materials and Methods

A cross-sectional descriptive study was conducted in 36 Romanian AICDs in 32 hospitals, representing 43% of intensive care departments in our country. Included hospitals were categorized into two groups: (a) tertiary hospitals (university and county hospitals) and (b) secondary hospitals (city hospitals).

The Hospital Survey on Patient Safety Culture (HSOPSC) questionnaire was used for this study [9]. The HSOPC survey is already used in 71 countries and has been translated into 32 languages [10]. The HSOPSC survey has 42 items grouped into 12 dimensions. For the 12 dimensions, an overall score of the elements of each dimension, called composite items, was calculated, and the final rank of the dimensions was reached. To calculate the score for a given composite of the safety culture, the scores on all items included in the composite were averaged. In order to calculate the correct score, recoding was performed by reversing for items with negative formulation. If we obtain a disagreement (values of 1 or 2) on a negatively worded item, we have obtained a positive response. Negatively worded items are identified in the Hospital Survey on Patient Safety Culture [9]. The translated questionnaire was pretested and verified for accuracy and clarity by 10 specialists in quality management. A 5-point Likert response scale was used (“1” corresponds to “not agree” and “5” to “fully agree”). On the HSOPSC’s 5-point Likert scale for assessing patient safety culture, scores represent employees’ perception of whether a safety culture exists within the organization. The 1- strongly disagree and 2- disagree scores represent a negative perception of the presence of individual and group values, attitudes, perceptions, competencies, and behavior patterns that determine commitment to patient safety. They also represent evidence for the existence of real obstacles for ICU and Anesthesia professionals to properly perform their duties correctly to provide sure and safe care. Scores 4 and 5 are obtained where perceptions are associated with a positive safety culture and are characterized by communications based on mutual trust, shared perceptions of the importance of safety, and confidence in the effectiveness of preventive measures. Neutral perceptions are associated with a lack of barriers to the proper performance of duties, but further efforts are needed for attitudes and behavioral patterns to be associated with a culture of patient safety.

A cover letter with further details about the study’s goals, the voluntary nature of participation, and assurances that participants’ identities and confidentiality would be maintained was provided to invited participants. A 5-day window was agreed upon for completing the survey between November 2018 and June 2019.

The target population was all categories of staff working in AICDs in Romanian hospitals. Of all AICDs, 32 hospitals decided to take part in the survey. Participation was voluntary and was finally distributed to 1200 healthcare workers of public hospitals. No data on temporary staff were collected for this study. Only employees of health facilities were covered in the study. Employee perceptions of “use of temporary staff for better patient care” is a composite element of the “Staffing” dimension for assessing how staff allocation corresponds to the time needed to provide the best patient care. The perception of the survey participants was that the use of this staffing category is less used in ICUs in Romania. As this study was non-interventional, no Ethical Committee approval was required by our regulations.

Data management and statistical analyses were performed using IBM SPSS Statistics 27. Questionnaires were excluded if either blank questionnaires, those containing only responses with demographic data, or those answering each question using the same answer to all questions were excluded. Therefore, 17 questionnaires were excluded from the final analysis. Inferential tests were considered significant at α = 0.05. Inferential analyses were conducted after checking assumptions of normality. Exploratory Pearson’s correlations assessed relationships between key items. Multiple regression analyses were performed to describe associations between patient safety culture, represented by the frequency of events reported, and composite items.

## 3. Results

In total, 927 questionnaires were returned. The response rate was 77.3%, of which 749 (80.8%) were from tertiary and 178 (19.2%) from secondary hospitals. The majority of respondents (89.1%) worked in an Intensive Care Unit (ICU), while 10.9% worked in the general anesthesia area.

According to Table 1, most respondents (26.2%) had 1–5 years of experience, followed by those with 6–10 years of experience (19.5%). The number of hours worked each week was heterogenous. The majority (73%) of respondents worked between 40 and 59 h per week, whereas 16.3% and 5.9%, respectively, of respondents worked between 20 and 39 or between 60 and 79 h per week. In total, the working hours per week determined for a six-month period was no greater than 48 h per week.

Table 2 and Table 3 shows participants’ perceptions to each dimension concerning patient safety culture summarized for both hospital categories.

**Figure 1:**
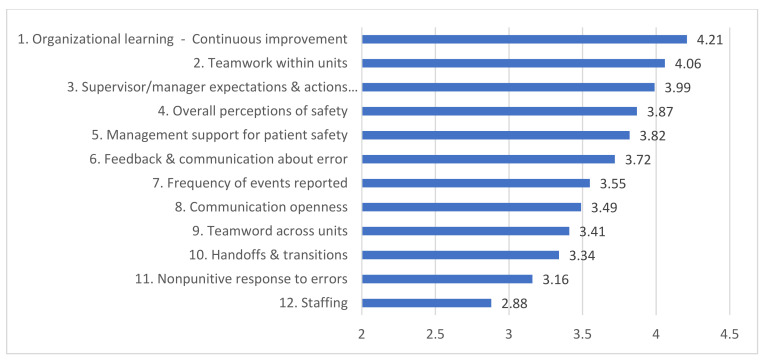
Figure shows ranking of the 12 dimensions of interest based on the data from the current study performed in Romania.

For nine dimensions, the mean scores were between “neutral” attitude and “agreement”. Two dimensions, “Teamwork within units” (4.06 ± 0.72) and “Organizational learning—Continuous improvement” (4.21 ± 0.56) (Figure 1), obtained the highest scores, being associated with a positive attitude of “agreement”. “General perceptions of patient safety” (3.87 ± 0.76) received good scores for both hospital categories (n.s.).

For seven dimensions, there were significant differences between tertiary and secondary hospitals and higher scores were reached by secondary hospitals. Employees in AICDs of secondary hospitals perceived “Teamwork within units” (4.36 ± 0.51) much better than those in tertiary hospitals (3.96 ± 0.72).

The linear regression model showed significant correlations between “Frequency of events reported” and all items (*p* < 0.001), but not with Staffing (*p* = 0.78), when all hospitals were taken into consideration.

In the analysis of the perception of the different professional groups, significant differences were identified according to the respondents’ years of experience. The perceptions of professionals with more than 5 years’ experience were analyzed compared to those who had less than or a maximum of 5 years’ experience in anesthesia and intensive care departments. In the case of two dimensions, we obtained significant differences (*p* < 0.01). “General perception of patient safety” (4.14 ± 0.72) was rated higher in those with less than or equal to 5 years’ experience than in those with more experience—more than 5 years (3.37 ± 0.58). However, in the case of “Handoffs & transitions” the perception of those with more experience was that this process meets the safety requirements better (3.79 ± 0.54) than those with 5 years of experience or less (2.96 ± 0.764).

## 4. Discussion

In Romania, the European Commission’s recommendations to promote patient safety culture have been implemented since 2009 [11] to approve quality and patient safety standards for national hospital accreditation [12]. Since 2018, specific standards for anesthesia have also been approved according to European recommendations in Romania. In addition to all these efforts at a national level, thus far, no tool has been used to assess the culture of patient safety or measure the effectiveness of promoted policies and activities.

Research on patient safety culture showed that adverse events are linked to immature safety cultures, unconsolidated teamwork, fragile interdepartmental working relationships, and increased cognitive demands such as “difficulties on decision making” and “simultaneous management of many tasks” [13]. Anesthesia and intensive care departments have a high workload and tackle complex tasks that have the potential to negatively impact patient outcome [14].

Patients with complex and severe illnesses, the organizational and managerial environment, as well as the interdependence of healthcare workers within units have an impact on patient safety, and thereby on medical errors in ICUs. According to the latest definition, medical errors represent “any preventable event that may cause or lead to inappropriate medication use or patient harm while the medication is in the control of the healthcare professional, patient, or consumer” [15]. In order to increase or ensure patient safety, efforts must be put in place to avoid or minimize errors. Therefore, it is important to analyze the organization’s patient safety culture to learn and improve. A baseline evaluation was initiated using HSOPSC in Romania, as it is considered “one of the most rigorous instruments tested” [16], with good and validated psychometric properties [17].

We also compared our results with results using the same questionnaire in France and Austria [18,19]. Our research group chose to compare the results of this study with published results from Austria and France because of similarities in implementing and evaluating patient safety culture, which have been reported in many recent publications, and because some elements of their procedures are also implemented in Romanian practice due to the requirements of the National Authority for Quality Management in Healthcare in Romania. These similarities are more generalized at the level of public institutions in Romania with respect to Austria and France in the field of quality and safety in healthcare. Another reason in favor of France is that the health insurance system in France is similar to the one in Romania. In both countries, the system is administered by the state through compulsory insurance.

On the one hand, the results of this study showed that the overall perception of safety culture is good (3.87 ± 0.76) compared to that of France (3.21) and Austria (3.68) [18,19]. On the other hand, the perception of nonpunitive response to errors obtained lowest scores. For example, in France, it scored the lowest (2.94) [18]. Similarly, a study from the Czech Republic found that there was a higher frequency of reported events in cases in which feedback and communication about errors were accepted and organizational learning and continuous improvement were ensured [20]. This finding is also supported by our results, in which feedback and communication about errors showed high scores.

The current law in Romania obliges hospitals to immediately report incidents and adverse events associated with medical errors within 24 h. As this requirement is known and continuously promoted within Romanian hospitals, the dimension “organizational learning and continuous improvement” in the current survey showed the highest score (4.21 ± 0.56).

Similar to other studies [18,19], an interesting aspect in our study was the results showing as strong dimensions of safe care; those related to the expectations and actions of the manager/supervisor promoting patient safety (3.99 ± 0.71) were appreciated by the respondents, who stated that their manager takes patient safety issues into account and considers staff recommendations on ways to improve patient safety.

On the other hand, these data did not correlate with the low frequency of reporting adverse events. Data on the low frequency of error reporting in our study may also show that the mechanism of written reporting is not routinely used.

The dimension of “Management support for patient safety” obtained high scores in tertiary (3.70 ± 0.83) as well as secondary (3.97 ± 0.89) hospitals. On the contrary, a Swedish study found that “patient safety management support” was low, while “manager’s expectations and actions that promote safety” were ranked highly. This may lead to the assumption that respondents were quite satisfied with the measures employed in their departments. Nevertheless, they thought that top managers could support even more patient safety initiatives [21].

In general, respondents preferred to work in teams and to support each other to solve tasks in order to provide their patients with better care. Nevertheless there were significant differences between both hospital categories. Regarding “interdepartmental collaboration” lower scores were accompanied by lower scores for “transfers and documentation”.

Lowest scores were seen for the staff dimension, which was defined as the extent to which staff allocation corresponded to the volume and time required to provide the best care to patients. This dimension was comparable to results of other studies in Europe [22,23,24]. A linear regression model showed that has no influence on the frequency of events reported [23]. Respondents’ low perception may also be caused by physical and mental fatigue and insomnia, which increase the likelihood of committing errors. In our study, it is noteworthy that tertiary hospitals use slightly more temporary staff for patient care (*p* = 0.005) than secondary hospitals do. They also work more in a so-called “crisis mode”, “trying to do too much and too much fast” (*p* = 0.001) compared to secondary hospitals. Thus, it is recommended to improve working time and conditions to reduce the burden of tasks. It is necessary to provide predictability and clearly defined roles and provide employees with feedback to create a culture of safety [24]. Furthermore, revision of regulations and staff re-allocation is needed. Specific human resources strategies, different between tertiary and secondary hospitals, are needed to meet the needs of safety, volume, and stress concerning tasks of AICDs.

The “Staffing” dimension is an assessment of employees’ perceptions of how human resources are managed at the organizational level. As the dimension that needs the most improvement, it means that human resource management strategies need to be developed to properly address the appropriate and clear allocation of roles and responsibilities according to the skills of employees and the needs of patients. Clear communication of expectations and a better understanding of teamwork can be achieved by applying effective communication techniques and strategies. Institutional communication can enhance the patient safety culture by creating an environment of learning from mistakes and developing a climate of trust.

In the meantime, the COVID-19 pandemic placed additional pressure on the healthcare system, especially on ICUs. Any vulnerability regarding the organization, staff deficiencies, compliance with rules and staff allocation in this context can only increase pressure and requires an approach to organizational models based on predictability and special programs to increase secure environments with a direct impact on patient safety [25]. Low nurse staffing was associated with poor organizational climate. Staff in these areas also reported twice as many errors as those in well-staffed equipped units. Therefore, healthcare systems should take responsibility for reorganization. Other processes and managerial tasks are needed to change attitudes at an organizational level, so that exhaustion can be considered an unacceptable risk, rather than a sign of dedication. Healthcare professionals should no longer experience fear when they report errors or adverse events in their practice [26]. To develop a patient safety culture, employees need to feel protected. Staff who report problems need to trust that they will not be judged unfairly by management. Senior employees and management must be concerned with identifying factors that contribute to problems. Identifying the causes of errors through an audit and notifying those involved of the findings can help improve trust and culture. Developing communication and teamwork skills through simulation techniques, briefing, and debriefing after certain clinical situations can help to encourage error reporting within a climate of tolerance and trust.

There are several limitations within this study. First of all, the study did not include all AICD in Romania, as only 43% took part. Although a large number of AIDCs from tertiary and secondary public hospitals in Romania were included, a selection bias may be possible. Another limitation refers to the frequent occurrence of no answer being given in self-administered questionnaires. An additional limitation of this research was the discrepancy between ICU and anesthesia staff (with predominance of ICU staff), although all staff belong to the same department. However, this discrepancy may have influenced our results since, in anesthesia, risks, communication, and patients’ risks and problems may be different from those in the ICU. There are still hospitals in Romania where staff is rotated from the ICU to operating rooms on regular bases. This was one of the reasons for asking all staff to respond the questionnaire.

Another possible limitation of this study is the usage of a Likert-type scale for measuring the dimensions. Although Likert-type scales remain prevalent in health studies due to their simplicity and ease of usage, acquiescence bias can potentially alter results. Acquiescence bias refers to the tendency of respondents to disproportionately choose ‘’agree’’ on the Likert questionnaire, possibly because of the social norm to be agreeable [27]. Acquiescence can introduce errors into data, as survey responses to acquiescence-prone measures conflate individuals’ true attitudes and behaviors with agreeableness. At its most pernicious, this can lead survey researchers astray, inducing correlations between similarly worded items that may be designed to tap unrelated constructs and hence resulting in systematic errors.

## 5. Conclusions

The recent study revealed that organizational learning, continuous improvement, teamwork, and management support for patient safety are highly present in Romanian AICDs. Communication across departments and fostering a non-punitive culture in the event of negative events are two areas in need of further improvement. A distinct strategy for staffing between tertiary and secondary hospitals is also needed. It is crucial to focus on staffing, as it might have a major impact on decreasing errors and increase the overall culture of safety. To increase the frequency of events reported as an indicator to improve patient safety, specific measures are needed. Of these, the promotion of feedback and open communication seems to be most important.

## Figures and Tables

**Table 1:** Demographic data (*n* = 927).

	(%)
**Professional Group**	
Nurse	64.7
Social worker	1.0
Senior physician	9.9
Resident physician	1.8
Technician	0.1
Kinetotherapist	1.1
Department secretary	0.1
Cleaning staff	9.6
Other (e.g., porters)	8.2
Not mentioned	3.5
**Years of Experience in AICD**	
Less than 1 year	7.6
1–5 years	26.2
6–10 years	19.5
11–15 years	17.6
16–20 years	12.2
21 years or older	14.0
Not mentioned	2.9
**Worked Hours per Week**	
Less than 20	1.1
20–39	16.3
40–59	73.0
60–79	5.9
80–99	0.2
Not mentioned	3.5

**Table 2:** Survey results.

	Dimensions of Patient Safety Culture	Mean	SD	Median	Rank
**1**	Communication openness	3.49	0.79	3.67	8
**2**	Feedback & communication about error	3.72	0.93	4.00	6
**3**	Teamwork within units	4.06	0.72	4.00	2
**4**	Nonpunitive response to errors	3.16	0.94	3.00	11
**5**	Organizational learning—continuous improvement	4.21	0.56	4.33	1
**6**	Supervisor/Manager expectations & Actions promoting patient safety	3.99	0,71	4.00	3
**7**	Staffing	2.88	0.71	2.75	12
**8**	Teamwork across units	3.41	0.78	3.50	9
**9**	Handoffs & transitions	3.34	0.68	3.25	10
**10**	Management (leadership) support for patient safety	3.82	0.84	4.00	5
**11**	Frequency of reported events	3.55	1.2	3.67	7
**12**	Overall perception of safety	3.87	0.76	4.00	4

**Table 3:** Comparing employees’ perceptions of AICDs from tertiary hospitals (*n* = 749) to employees’ perceptions of AICDs from secondary hospitals (*n* = 178).

	Composite Items		Mean	SD	Median	*p*-Value
1	Teamwork within units	Tertiary hospitals	3.96	0.72	4.00	*p* < 0.001
		Secondary hospitals	4.36	0.51	4.38	
2	Expectations and actions of the manager/supervisor promoting patient safety	Tertiary hospitals	3.93	0.71	4.00	*p* < 0.001
		Secondary hospitals	4.14	0.71	4.25	
3	Organizational learning—continuous improvement	Tertiary hospitals	4.15	0.57	4.00	*p* < 0.001
		Secondary hospitals	4.35	0.40	4.33	
4	Management (leadership) support for patient safety	Tertiary hospitals	3.70	0.83	3.67	*p* = 0.001
		Secondary hospitals	3.97	0.89	4.00	
5	Overall perceptions of patient safety	Tertiary hospitals	3.82	0.75	4.00	*p* = 0.86
		Secondary hospitals	3.86	0.85	3.86	
6	Error feedback and communication	Tertiary hospitals	3.67	0.93	3.67	*p* = 0.005
		Secondary hospitals	3.91	0.93	4.00	
7	Openness to communication	Tertiary hospitals	3.49	0.79	3.67	*p* = 0.007
		Secondary hospitals	3.63	0.72	3.67	
8	Frequency of reported events	Tertiary hospitals	3.49	1.15	3.67	*p* = 0.07
		Secondary hospitals	3.37	1.36	3.67	
9	Teamwork across hospital units	Tertiary hospitals	3.35	0.81	3.5	*p* < 0.001
		Secondary hospitals	3.57	0.77	3.5	
10	Staffing	Tertiary hospitals	2.83	0.69	2.75	*p* < 0.001
		Secondary hospitals	3.22	0.76	3.25	
11	Handoffs & transitions	Tertiary hospitals	3.31	0.69	3.25	*p* = 0.017
		Secondary hospitals	3.39	0.63	3.5	
12	Nonpunitive response to error	Tertiary hospitals	3.10	0.93	3.00	*p* = 0.002
		Secondary hospitals	3.47	0.97	3.33	
